# Neurocognitive functions in children and adolescents with different enthusiasm for video games

**DOI:** 10.1192/j.eurpsy.2021.554

**Published:** 2021-08-13

**Authors:** G. Soldatova, A. Vishneva, A. Koshevaya

**Affiliations:** 1 Faculty Of Psychology, Lomonosov Moscow State University, Moscow, Russian Federation; 2 Clinical Psychology, center for speech pathology and neurorehabilitation, Moscow, Russian Federation

**Keywords:** neurocognitive development, adolescents, Children, video games

## Abstract

**Introduction:**

Video games are becoming increasingly popular among children (Lenhart et al., 2015). There is a lack of research that studies the impact of online games on children’s neurocognitive functions.

**Objectives:**

The aim is to study neurocognitive functions in children and adolescents playing and not playing online games.

**Methods:**

The sample comprises 100 children aged 5-10 years and 100 adolescents aged 11-16 years. The following neuropsychological indexes (Akhutina, 2016) are studied: programming and control, serial organization of movements, auditory and visual memory, left and right hemispheric functions, and neurodynamic component of mental activity. Wexler’s Awareness and Comprehension Tests were used to study verbal functions. The game activity are measured by social-psychological questionnaire.

**Results:**

Children who play online games have a serial organization of movements (smooth switching from one component of the program to another) (F=14,46, p<0,01) and a neurodynamic component (F=13,07, p<0,01), which are worse developed than children who do not play online games. Adolescents playing online games have better analytical (left hemispheric) functions (F=13,37, p<0,01), mathematical abilities (F=3,47, p=0,063), and Awareness subtest (F=3,47, p=0,065) scores than nonplaying adolescents.

**Conclusions:**

Children playing online games have lower results on neurocognitive functions directly related to motor development. Teenagers playing online games had higher scores in mathematical ability, analytical functions and awareness. The results indicate the need to develop an optimal time for digital gaming activities depending on the age of the child. The reported study was funded by RFBR, project No. 19-29-14181.

**Conflict of interest:**

The reported study was funded by RFBR, project 19-29-14181.

